# Pulmonary Manifestations of Primary Sjögren's Syndrome: Underlying Immunological Mechanisms, Clinical Presentation, and Management

**DOI:** 10.3389/fimmu.2019.01327

**Published:** 2019-06-12

**Authors:** Sarthak Gupta, Marcela A. Ferrada, Sarfaraz A. Hasni

**Affiliations:** National Institute of Arthritis and Musculoskeletal and Skin Diseases, National Institutes of Health, Bethesda, MD, United States

**Keywords:** primary Sjögren's syndrome, interstitial lung disease, ILD, pulmonary manifestations, pSS, lung involvement

## Abstract

Pulmonary involvement in primary Sjögren's syndrome (pSS) is an understudied entity with important clinical implications. Its prevalence has been reported in up to 20% of pSS patients. Pulmonary manifestations of pSS are diverse with involvement of airway and/or lung parenchyma. Histopathology of lung lesions suggests a predominance of submucosal mononuclear cell infiltration consisting predominantly of CD4+ T cells. Current understanding of the pathophysiology of lung disease in pSS suggests a similar process driving the pulmonary process as those in the salivary glands, with epithelial cells playing a critical role in the initiation, maintenance, and symptomatology of the disease. Clinical manifestations of lung involvement in pSS are as varied as the underlying pathology and can be vague and non-specific, thus delaying diagnosis. Management options depend on the underlying pathology but are generally limited due to lack of systematic randomized controlled trials. This review helps summarize our current understanding of lung involvement in pSS.

## Introduction

Primary Sjögren's syndrome (pSS) is a progressive systemic autoimmune disease primarily affecting females that is manifested by inflammatory lymphocytic infiltrate of exocrine glands like lachrymal and salivary that lead to the destruction of the tissue. It is characterized by dry mouth and eyes, parotid swelling, profound fatigue, widespread musculoskeletal pain, and polyarthritis (1). Its prevalence is estimated at 0.5−4%, making it one of the most prevalent multisystem autoimmune disease after rheumatoid arthritis (2–4). Apart from dry eyes and dry mouth, pSS has multiple systemic manifestations including polyarthritis, autonomic dysfunction, pancreatitis, vasculitis, renal involvement, lymphoma, fatigue, increased immunoglobulin, hypocomplementemia, and lung involvement (5).

Similar to other systemic autoimmune diseases, Sjögren's syndrome develops in genetically predisposed individuals, who are exposed to various environmental factors resulting in a dysfunctional immune system response to self-antigens. The impaired immune system cascades by activation of innate immune response along with activation of glandular epithelial cells that in turn leads to activation of B and T lymphocytes resulting in eventual damage of the target organs (6).

The lung manifestations of pSS are varied and include airway abnormalities and interstitial lung disease (ILD) (7). Even though it may be present in nearly a fifth of pSS patients, it is an understudied entity with important clinical implications. Patients with pulmonary involvement have decreased quality of life and increased mortality as compared with patients with pSS with no pulmonary involvement (8, 9) This review summarizes our current understanding of the pulmonary manifestations of pSS and includes clinical management of this entity.

## Prevalence of Lung Disease in Sjögren's Syndrome

The pulmonary manifestations of pSS are diverse with airway disease and ILD being the most predominant presentations. Additionally, the manifestations differ in severity and this could in part explain the wide variability in the reported prevalence of this entity. The prevalence of clinically significant pSS lung disease have been reported from 9 to up to 20% (10–12). However, the estimates of prevalence increase significantly (43–75%) on comprehensive evaluation with imaging modalities and pulmonary function tests, suggesting a wider subclinical presentation (13). Computed Tomography (CT) scans of pSS patients have shown lung changes in up to 34−50% of patients (13, 14). In one study, the annual incidence of pulmonary manifestation like ILD has been estimated at 10% at just 1 year of diagnosis and increases to nearly 20% by 5 years (15).

## Pathogenesis

The primary histological lesion of pSS is progressive focal lymphocytic infiltrate around the salivary and lachrymal ducts that gradually extends and replaces the physiological glandular epithelium leading to dry eyes and dry mouth. Mononuclear cells, enriched in CD4+ T-cells, are found to infiltrate these lesions (16). Interestingly, similar lesions have been seen in extraglandular organs like kidneys (17–19), and liver (20) and also, in lung lesions. Additionally, increased lymphocytic infiltration of salivary glands, quantified by focus score, has been shown to correlate with increased prevalence of airway and interstitial lung disease in pSS (21). All these findings suggest that the pathways leading to glandular and extraglandular manifestations of pSS are similar and involve development of autoimmunity to epithelial cells, and that epithelium plays a key role in pSS pathogenesis.

**Figure 1:**
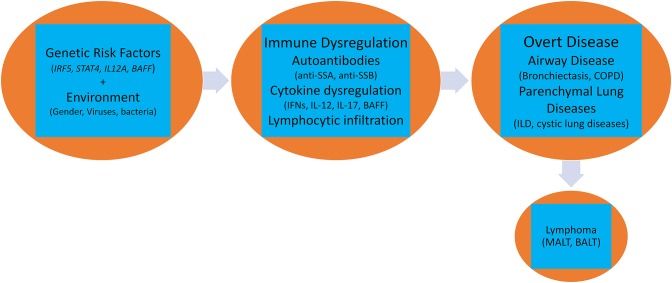
Pathogenesis of pulmonary involvement in Sjögren's syndrome.

A complex interaction of genetic, environmental, and hormonal factors has been implicated in the pathogenesis of pulmonary pSS. Activation of several biological pathways belonging to both innate and acquired immune systems such as Type I and II interferons (IFN) (22), aberrant T-regulatory activity (23), augmented function of helper T-cells (24), lymphoneogenesis with germinal center formation, and abnormal B-cell activation with clonal expansion of B-cells (25) has been reported (Figure 1).

However, it is still unclear why epithelial cells like those present in the airway or lung parenchyma are targeted in pSS. This could be an extension of the immune response that originated in the salivary glands and subsequently affects other epithelial cells in the body. Activation of B and T cell responses against shared common antigens like ion-transport channel, enzymes like carbonic anhydrase, or muscarinic receptors that are expressed in all epithelial cells have been reported in pSS (26, 27). Autoantibodies against M3 muscarinic receptor may also lead to compensatory increase in M3R expression resulting in cholinergic hyperresponsiveness as seen in some pSS with hyper-reactive airway disease (28).

Alternatively, studies have also implicated viruses (29) and bacteria (30) that have a specific tropism for epithelial cells as initial drivers of disease; however, a specific responsible agent has not been conclusively identified. For example, human T-lymphotropic virus type 1 (HTLV-1) is one of the pathogens reportedly attributed in pSS pathogenesis (31, 32). Anti-HTLV-1 antibodies are significantly more prevalent in pSS patients with airway disease than without (21). Interestingly, HTLV-1 lung infection, even in the absence of any connective tissue disease, has been associated with radiographic and pathological findings consistent with lymphocytic bronchiolitis and ILD (33–35). Additionally, p40^tax^, a regulatory component of HTLV-1 that has the ability to induce multiple host genes including pro-inflammatory cytokines like IFNγ, is highly expressed in BAL from patients with HTLV-1 bronchiolitis (36). BAL from these patients also have increased numbers of activated T-cells (35). The similarities in the lung manifestations in HTLV-1 infection and that seen in patients with pulmonary pSS suggests a possible role of HTLV-1 in pathogenesis of pSS lung.

Individuals with aberrations in innate immune molecules like surfactant protein-D (SP-D) may also develop altered immune responses to viral or bacterial pathogens. SP-D is a member of the collectin family, pattern-recognition molecules which bind to carbohydrate moieties presented on a variety of pathogens and help in neutralization and clearance of microorganisms (37, 38). It is primarily synthesized by alveolar type II cells and its receptor is expressed on endothelial cells, B-cells, and antigen presenting cells (APCs). SP-D has been found to be decreased in serum from pSS patients with bronchiectasis, suggesting a possible role in pathogenesis (39). Interestingly, active pulmonary inflammation as seen in ILD due to pSS leads to increased circulating SP-D and may be beneficial as a serum biomarker.

Epithelial damage, either due to pathogen or due to genetic predisposition, and altered immune responses result in apoptosis and could be responsible for release of autoantigens (40), which may in turn lead to epitope spreading and damage of other epithelial cells in the body. Notably, SSA, Sjögren's syndrome related antigen A, a key autoantigen in pSS, redistributes during apoptosis of glandular cells and can be presented along with major histocompatibility complex (MHC) class II molecules on membrane of these apoptotic cells, and on the apoptotic bodies to induce an antibody response (41, 42). The presence of autoantibodies to SSA long before clinical manifestation of pSS suggests a prolonged subclinical phase with multiple or recurrent hits. The strong association of anti-SSA antibodies to pulmonary pSS manifestations also suggests ongoing damage to the pulmonary epithelial cells as a possible driver of pulmonary pSS pathogenesis.

It is likely that the patho-biology driving the disease in salivary glands is also responsible for the lung manifestations, where eventually, release of autoantigens results in stimulation of intrinsic cellular responses with engagement of toll-like receptors (TLRs), like TLR3 (43) in epithelial cells, and TLR7 and TLR8 in plasmacytoid dendritic cells (pDCs), that results in local up-regulation of Type I IFNs and IL-12 (44–46). Increase in Type I IFNs induces release of B-cell activating factor (BAFF) (47) that stimulates B-cell activation and autoantibody production and eventual formation of immune complexes that can stimulate pDCs leading to further release of Type I IFN (48). IL-12 along with Type I IFNs stimulates both natural killer cells (innate) and Th1 cells (adaptive) that increase IFNγ production and mediate tissue damage (49). In transgenic mouse model, increased IL-12 led to characteristic changes seen in pSS lung like lymphocytic infiltrates around the bronchi, intraluminal debris, increased cell proliferation in the alveoli, and increased interstitial and alveolar macrophages. This suggests that IL-12 is a key pathogenic cytokine in pSS lung (50).

Epithelial cells, upon TLR stimulation, also produce T-cell homeostatic cytokines, like IL-7, that is an important mediator of T-cell activation (51), IFN-gamma-mediated Th1 response (52) and maintenance of pathogenic Th17 cells (53). These infiltrating CD4+ T cells (Th1 and Th17) show an activated phenotype and release pro-inflammatory cytokines like IFNγ (Type II IFN), IL-17 and IL-6. IFNγ is involved in epithelial cell activation, apoptosis, inflammation, and tissue damage (54). IL-17 has a pro-inflammatory effect on epithelial cells by induction of matrix metalloproteinases secretion, dysregulation of tight junction proteins, and support of ectopic lymphoid tissues (55). IL-6 promotes Th17 differentiation and is in turn important for the induction of IL-17 (55). CD4+ T cells also provide support to recruited B-cells, leading to further tissue damage. Subsequently, infiltrating T-cells organize with the B-cells to form germinal centers in affected tissue, a characteristic feature that is also seen in pulmonary pSS (56, 57).

Another evidence of lymphoproliferation in lung tissue comes from elevated beta-2-microglobulin that is released by lymphocytic tissue and elevated in serum from patients with pSS, particularly pulmonary pSS (58, 59). Lahdensuo and Korpela prospectively studied seventeen patients with pSS with no history of smoking to evaluate the correlation between pulmonary findings including chest CT and pulmonary function tests and serum beta-2-microglobulin as well as immunoglobulins including IgA, IgG, IgM. Interestingly they found nine patients with hyperinflation based on an elevated residual volume (RV)/total lung volume (TLC) ratio. Although, there was no significant differences between the serum immunoglobulins, when comparing patients with hyperinflation with those without, the mean level of serum beta-2 -microglobulin was significant elevated in patients with hyperinflation (60). The authors hypothesized that perhaps the elevation on beta-2-microglobulin may reflect the severity of lymphoproliferation and that high levels may imply that obstructive small airway disease is associated with advance lymphoproliferative stage of the disease. Furthermore, Pertovaara and collaborators also evaluated the significance of baseline beta-2-microglobulin concentration as a risk factor for pulmonary involvement in 19 patients with pSS (61). They found that baseline elevated beta-2-microglobulin levels correlated with the development of subtle restrictive changes in pulmonary function test. Interestingly they also found an elevation of ESR, IgG, and protein concentrations suggesting an immunological activity in patients that had elevated beta-2-microglobulin and develop mild restrictive changes.

## Histology

The pathology of Sjögren's lung disease varies with the location and type of involvement. Airway disease can be due to hyper-reactive airways, damage to the exocrine glands or due to cell infiltration and can affect trachea, bronchi, or bronchioles. Extraglandular mononuclear cell infiltration of bronchial and bronchiolar submucosa, largely comprising CD4+ T cells, have been described in both large and small bronchi (16, 62, 63). Lymphocytic infiltrates have even been reported in broncheoalveolar lavages (BAL) from asymptomatic patients who have normal radiographs (16, 64). Bronchiolitis is the most common airway disease in pSS patients with frequency reported to be between 12 and 24% (62, 65). Different types of bronchiolitis have been identified with a majority being follicular bronchiolitis (66), which is characterized by hyperplastic lymphoid follicles with a reactive germinal center that is distributed along the broncho-vascular bundles (7, 67). Other bronchiolitis, like chronic bronchiolitis, obliterative bronchiolitis, and panbronchiolitis have also been reported (66) and present with a spectrum of histological changes indistinguishable from other etiologies like lymphocytic infiltration of small airways, bronchiolar smooth muscle hypertrophy, obstruction of bronchioles with mucus or even complete obliteration of bronchioles with scarring.

Among the ILDs seen in patients with pSS, non-specific interstitial pneumonitis (NSIP) is the most frequent pathological subtype and has been reported to be present in up to 45% of histopathology available from patients (11, 64, 65, 68, 69). NSIP usually presents on histology with varying interstitial inflammation and fibrosis with preserved lung architecture (7, 70). BALs have also shown lymphocytic alveolitis in pSS patients (64). Interestingly pSS patients who have autoantibodies to SSA, a key autoantigen in pSS, have higher risk of developing ILD (71, 72). Although, one recent study did not find that association, with similar antibody levels in patients with or without anti-SSA antibodies (69). Additionally, patients with lymphocytic alveolitis have increased gammaglobulins, and an increased prevalence of rheumatoid factor and antinuclear antibody (ANA). Areas of interstitial fibrosis in patients with NSIP may result in traction bronchiectasis due to enlarged air spaces resulting in a CT pattern which helps distinguish it from usual interstitial pneumonia (7).

Usual interstitial pneumonia (UIP) is another type of ILD reported in up to 16–33% of pSS patients with ILD (11, 69, 70). Similar to UIP in other diseases, it is characterized by honeycombing on lung biopsy with histopathological findings of sub-pleural and peripheral lesions, patchy interstitial fibrosis, scattered fibroblast foci, and mild interstitial inflammation with patchy alveolar infiltrates of lymphocytes, plasma cells and lymphoid follicles with germinal centers (57). Another type of ILD reported in pSS patients is lymphocytic interstitial pneumonitis (LIP) accounting for about 15% of ILD lesions in this disease (11, 69). It shows diffuse abundance of polyclonal lymphocytes and plasma cells in the pulmonary parenchyma with lymphoid follicles and even germinal centers. Organizing pneumonitis is another ILD reported in up to 7% of patients with histological findings similar to other etiologies with patchy involvement of pulmonary parenchyma by fibromyxoid plugs of granulation tissue with sub-pleural or peri-bronchovascular distribution (11).

Patients with Sjögren's syndrome have an increased risk of non-Hodgkin lymphoma, specifically marginal zone B-cell lymphoma, and mucosa associated lymphoid tissue (MALT) lymphoma (73, 74). Primary pulmonary lymphoma, also known as bronchial-associated lymphoid tissue (BALT) lymphoma has been reported in 1–2% of patients with pSS (75). There is no organized lymphoid tissue in the normal pulmonary parenchyma but inflammatory conditions such as pSS and chronic exposure to various antigenic stimuli can lead to lymphocytic hyperplasia which may transform into a BALT lymphoma (76, 77). These lymphoma originate from marginal zone and are characterized by infiltrate of lymphoepithelial cells invading bronchial epithelial tissues (77). These are usually negative for infectious agents like human herpesvirus-6 or Epstein-Barr virus. Other lung findings reported, however rare, are pulmonary amyloidosis, pulmonary embolism and pulmonary hypertension.

## Clinical Manifestations

Clinical manifestations in pSS can be non-specific and patients can present with dyspnea (62%), cough (54%), sputum production (14%), chest pain (11%), and/or fever (7%). Nearly three fourth of patients have positive anti-Ro/SSa and one third have anti-La/SS-B antibodies (11). Interesting, in a subset of patients, pulmonary involvement can be the initial manifestation of the disease and can even precede the diagnosis. In a prospective study that included 77 newly diagnosed pSS patients, 13 patients (16.8%) presented with ILD as the first presenting symptom (78).

Both large and small airways, and lung parenchyma can be affected in pSS. Patients can also have lymphoproliferative disorders as well as other abnormalities such as cystic lesions. These lesions can exist in isolation or coexist in the same patient. The prognosis of Sjögren's lung disease varies on the type of pulmonary involvement. Cystic lesions are usually associated with good survival and few complications as compared with ILD or lymphoid interstitial pneumonia that carries a poor prognosis in terms of not only survival but also quality of life.

### Airway Disease in pSS

**Figure 2:**
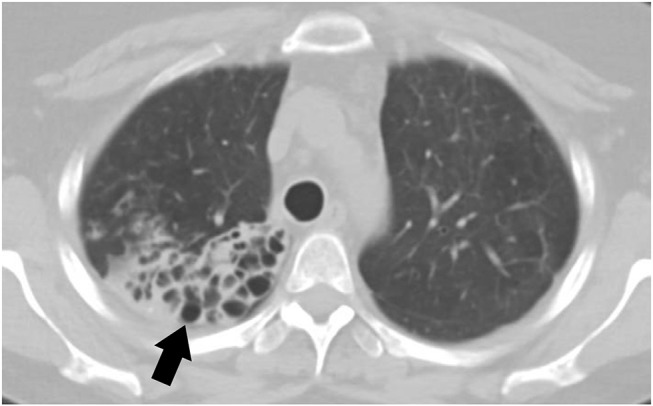
CT scan of a 44-year-old female with primary Sjögren's syndrome demonstrating bronchiectasis on the right lower lobe.

Small airway disease is the most commonly recognized pulmonary disorder among symptomatic pSS (79). Airway involvement can include bronchiectasis, bronchiolitis, and hyper-reactive airways. The prevalence of bronchiectasis in pSS is up to 10% with common involvement of the inferior lobes (Figure 2). Patients with bronchiectasis are generally older at diagnosis of pSS and have a greater frequency of hiatal hernia when compared to controls. Additionally, patients with bronchiectasis have a lower prevalence of anti-Ro/SS-A antibodies but increased presence of anti-smooth muscle antibodies (anti-SMA) (80). Clinical symptoms are variable and can be non-specific; for example, patients can complain of dry cough, or isolated dyspnea. In rare occasions patients can also have hemoptysis likely due to exacerbation of bronchiectasis. Patients with bronchiectasis also had increased respiratory infections and pneumonia (80).

Hyper-reactive airway is a common presentation of airway involvement in pSS. A small study where 15 patients were followed prospectively found a decline in pulmonary function test (PFT) parameters like diffusion capacity (DLCO), total lung volume (TLC), forced vital capacity (FVC), functional residual capacity (FRC), and expiratory midflows (FEF_50_) demonstrating distal airway involvement in those patients (81).

Chronic obstructive pulmonary disease (COPD) is common in patients pSS, including patients with no previous history of smoking (82). This was demonstrated in a study of 51 patients with pSS from which 30% of patients with pSS that never smoked fulfilled the global initiative for chronic obstructive lung disease criteria for COPD (83). Another study that included 41 female patients with pSS also demonstrated a high incidence of COPD in this particular population. Patients had a baseline pulmonary function test as well as high resolution chest CT scan and were followed prospectively for 11 years. At baseline, 7% of pSS patients were diagnosed with definite or suspected COPD. At follow-up, 37% of pSS patients fulfilled COPD criteria (84).

### Interstitial Lung Disease

The main symptoms of patients with ILD are cough and shortness of breath. It appears to be more frequently associated with positive anti-SSA, low levels of C3, elevated rheumatoid factor and C-reactive protein (69, 71, 72). Interstitial lung disease can also be present in different patterns with the most common being NSIP (33%), followed by UIP (23.8%), and LIP (9.5%) (69, 85). Interestingly, patients with ILD have more Raynaud's and gastrointestinal manifestations as compared with patients with pSS and no ILD. When compared to UIP patients with idiopathic pulmonary fibrosis (IPF), pSS patients with UIP tend to be older, predominately female, with enlarged mediastinal lymph nodes and bronchial thickening on HRCT. Reassuringly, pSS patients with UIP tend to have a better response to immunotherapy and thus a better prognosis as compared to patients with IPF (57).

### Cystic Lung Disease

Cystic lung disease has been noted in up to 20% of patients with Sjögren's syndrome. Cysts are mostly bilateral, with the majority located in the middle lung area, and have a benign course. The majority of the patients have no symptoms and do not develop a specific lung disease or lymphoproliferative disorder. Sjögren's patients with cystic lung disease tend to have increased prevalence of anti-SSB antibodies (86).

### Pulmonary Lymphoma

Different types of lymphomas have been associated with pSS including non-Hodgkin's lymphoma (NHL), marginal zone B cell lymphoma, and mucosa associated lymphoid tissue (MALT) that can present with symptoms of cough and slowly progressive dyspnea (75).

### Lymphocytic Interstitial Pneumonitis (LIP)

Common symptoms in LIP include cough and dyspnea, and physical findings can include coarse basal crackles. The clinical course is variable from complete resolution without treatment to progression and possible death or transformation to lymphoma (87).

## Evaluation

The European League Against Rheumatism (EULAR)—Sjögren's Syndrome (EULAR-SS) task force defined lung involvement in pSS based on a combination of three different parameters: clinical symptoms, pulmonary function test findings, and high-resolution chest computer tomography abnormalities. For example, EULAR SS Disease Activity Index (ESSDAI) considers pulmonary involvement in a patient with respiratory symptoms and normal radiology findings or patients with no symptoms but abnormal imaging or PFT's. The level of lung disease activity has been defined as low, moderate, or high (11) (Table 1). Symptomatic patients should be further evaluated for airway and parenchymal involvement with pulmonary function test as well as high resolution CT scan.

**Table 1:** Definition of pulmonary involvement in pSS by EULAR-SS Task Force.

**Low**	**Moderate**	**Severe**
Dry cough (-) Imaging	>70% DLCO >40%	DLCO <40%
Asymptomatic	80% <FVC >60%	FVC <60%
	ILD by Chest CT	ILD by Chest CT + NYHA III-IV

## Management

Treatment of pulmonary manifestation in pSS depends on the type of involvement. Patients with bronchiectasis can be treated with nebulized hypertonic saline to help with the secretion, as well as bronchodilator, as needed. Isotonic saline can help improve throat dryness seen in patients with pSS as well as voice production (88). On the other hand, treatment with anti-inflammatory medications have not been proven successful in treating patients with hyper-reactive airways (89).

Corticosteroids and other immunosuppressants such as azathioprine, mycophenolate, and cyclophosphamide have been studied in ILD due to connective tissue diseases and shown to have modest benefit (72, 90–94). However, these studies have very small numbers of patients with pSS; there still are no large clinical trials evaluating the effectiveness of immunosuppression in the treatment of pSS patients with ILD. Rituximab, a chimeric monoclonal antibody that binds to cell surface protein CD20 that is primarily and widely expressed on B-cells and triggers B-cell death, was evaluated for ILD treatment in one small open-label study of 10 patients. The patients were followed for 6 months after rituximab treatment and reported to have some improvement on the DLCO, fatigue, shortness of breath, or cough, as well as CT findings. This is a promising study that will need to be repeated with more patients and longer follow-up (95). LIP may respond to corticosteroids although the response is unpredictable.

With the critical role of BAFF and B-cells in the pathogenesis of pSS, targeting this molecule in pulmonary pSS using belimumab, an FDA approved anti-BAFF monoclonal antibody, may be beneficial. Additionally, targeting B-cells with a combination of rituximab and belimumab is currently being explored for pSS and may be considered for patients with severe lung involvement (trial NCT02631538).

Targeting T-cells by using abatacept, a CTLA-4 monoclonal that modulated T-cell co-stimulation, has shown some benefit in pSS with decrease in lymphocytic foci, reduction in local T-cells and gammaglobulins, and could be beneficial in pulmonary pSS (96).

Direct inhibition of inflammatory cytokines may be of potential benefit in pulmonary pSS too. IL-17 has been shown to play an important role in pathogenesis of pSS and can be targeted with monoclonal antibodies like secukinumab (97, 98) and iIxekizumab (99) that are already approved for psoriasis and psoriatic arthritis. IL-6, another pro-inflammatory cytokine attributable to pulmonary pSS, may also be of interest and RCTs looking at its role in pSS are currently ongoing (trial NCT01782235).

With the key role of IFNs in the pathogenesis of pSS, targeting them may be beneficial in pulmonary pSS. Anifrolumab is a monoclonal blocking type I IFN receptor (IFNAR1) currently being evaluated in SLE (100) and could be a potential agent for use in pulmonary pSS. Additionally, blocking downstream signaling of IFN and other pro-inflammatory cytokines signaling through use of janus kinase (JAK) inhibitors, the so called JAKinibs, may have wider benefit. Recent evidence suggests it may also influence BAFF signaling, thus modulating B cell responses (101). JAKinibs like tofacitinib have been recently approved for use for rheumatoid arthritis, psoriatic arthritis, and ulcerative colitis, and are being explored in SLE and pSS (102). There is some evidence that JAKinibs may be of benefit in chronic obstructive pulmonary disease (103) and idiopathic pulmonary fibrosis (104). It would be important to explore their role in pulmonary pSS.

## Conclusion

Pulmonary involvement in pSS is a prevalent, yet underexplored, clinical entity. Clinical manifestations are varied and can present with airway or lung parenchymal involvement. Accordingly, initial symptoms can be vague and non-specific. Histologic evidence suggests a common pathway that leads to glandular and lung pathogenesis with a key role played by epithelial cells. Patients with pSS should be systematically evaluated for pulmonary involvement and managed according to their presentation. Larger, systematic, randomized clinical trials are needed to evaluate various aspects of immunosuppression in pulmonary pSS. Based on our understanding of the biology of the disease, drugs targeting B-cells, like rituximab (anti-CD20) or belimumab (anti-BAFF); or those targeting T-cells, like abatacept (CTLA-4 Ig), could be beneficial. Direct targeting of pro-inflammatory cytokines like IL-17, IL-6, and Type I IFNs could also be beneficial. Additionally, small-molecules that block JAK signaling may offer a wider beneficial effect.

## Author Contributions

SH: concept, review, edit and supervision. SG and MF: draft and revisions. Both SG and MF contributed equally to the draft.

### Conflict of Interest Statement

The authors declare that the research was conducted in the absence of any commercial or financial relationships that could be construed as a potential conflict of interest.
